# Role of serum biomarkers in the diagnosis of infection in patients undergoing extracorporeal membrane oxygenation

**DOI:** 10.1186/cc10633

**Published:** 2012-03-20

**Authors:** M Pieri, T Greco, AM Scandroglio, M De Bonis, G Maj, L Fumagalli, A Zangrillo, F Pappalardo

**Affiliations:** 1 Istituto Scientifico San Raffaele, Milan, Italy; 2 Istituto Scientifico San Raffaele Turro, Milan, Italy

## Introduction

Although rates and causal organisms of infections occurring in patients on extracorporeal membrane oxygenation (ECMO) have already been described [1], diagnosis of infection itself is challenging in clinical practice. In addition, a significant heterogeneity in infection surveillance practice patterns among ELSO centers has recently been reported [2]. The aim of the study was to analyze the role of C-reactive protein (CRP) and procalcitonin (PCT) in the diagnosis of bacterial and fungal infection in critically ill patients requiring ECMO, and to assess the difference between venovenous (VV) and venoarterial (VA) ECMO setting.

## Methods

A case-control study on 27 patients. We analyzed serum values of PCT and CRP according to the presence of infection.

## Results

Forty-eight percent of patients had infection. Gram-negative bacteria were the predominant pathogens (54%), and Candida was the most frequent isolated microorganism overall (15%). PCT had an AUC of 0.681 (*P *= 0.0062), for the diagnosis of infection in patients on VA ECMO, but failed to discriminate infection in the VV ECMO group (*P *= 0.14). The AUC of CRP was 0.707 (*P *≤0.001) in all ECMO patients. In patients receiving VA ECMO, PCT had good accuracy with 1.89 ng/ml as the cut-off (SE = 87.8%, SP = 50%) and CRP as well with 97.70 mg/l as the cut-off (SE = 85.3%, SP = 41.6%). PCT and CRP tests in parallel had SE = 87.2%, and SP = 25.9%. Four variables were identified as statistically significant predictors of infection: PCT and CRP tests in parallel (OR = 1.184; *P *= 0.0008), age (OR = 0.980; *P *≤0.001), presence of infection before ECMO implantation (OR = 1.782; *P *≤0.001), and the duration of ECMO support (OR = 1.056; *P *≤0.001).

## Conclusion

Both traditional and emerging inflammatory biomarkers can help in the diagnosis of infection in patients receiving ECMO. Indeed, we demonstrated for the first time that PCT is a reliable infection marker in patients undergoing VA ECMO. We suggest routine concomitant PCT and CRP assay with definite cut-off values as a new test to identify infection in patients undergoing VA ECMO.
